# Predicting A-to-I RNA Editing by Feature Selection and Random Forest

**DOI:** 10.1371/journal.pone.0110607

**Published:** 2014-10-22

**Authors:** Yang Shu, Ning Zhang, Xiangyin Kong, Tao Huang, Yu-Dong Cai

**Affiliations:** 1 The Key Laboratory of Stem Cell Biology, Institute of Health Sciences, Shanghai Institutes for Biological Sciences, Chinese Academy of Sciences, Shanghai, P.R. China; 2 Department of Biomedical Engineering, Tianjin Key Lab of BME Measurement, Tianjin University, Tianjin, P.R. China; 3 Department of Genetics and Genomic Sciences, Icahn School of Medicine at Mount Sinai, New York, New York, United States of America; 4 Institute of Systems Biology, Shanghai University, Shanghai, P. R. China; University of Alberta, Canada

## Abstract

RNA editing is a post-transcriptional RNA process that provides RNA and protein complexity for regulating gene expression in eukaryotes. It is challenging to predict RNA editing by computational methods. In this study, we developed a novel method to predict RNA editing based on a random forest method. A careful feature selection procedure was performed based on the Maximum Relevance Minimum Redundancy (mRMR) and Incremental Feature Selection (IFS) algorithms. Eighteen optimal features were selected from the 77 features in our dataset and used to construct a final predictor. The accuracy and *MCC* (Matthews correlation coefficient) values for the training dataset were 0.866 and 0.742, respectively; for the testing dataset, the accuracy and *MCC* were 0.876 and 0.576, respectively. The performance was higher using 18 features than all 77, suggesting that a small feature set was sufficient to achieve accurate prediction. Analysis of the 18 features was performed and may shed light on the mechanism and dominant factors of RNA editing, providing a basis for future experimental validation.

## Introduction

RNA editing is a post-transcriptional RNA process that provides RNA and protein complexity for regulating gene expression in eukaryotes. After editing, RNAs include changes in their nucleotide sequence, leading to non-synonymous codon changes, modulation of alternative splicing patterns, introduction/removal of stop codons and modification of regulatory RNAs [1]–[2]. Five types of RNA editing have been elucidated [3]. In mammals, especially primates, the most common form of editing is the conversion of adenosines to inosines, which is catalyzed by a family of enzymes called adenosine deaminase acting on RNA (ADAR) [4]. ADAR enzymes are encoded by three dependent genes, ADAR1-3, which are located on chromosomes 1, 21, and 10, respectively. Hartner and his colleagues knocked out the *Adar1* gene from mice and found that *Adar1* ^−/−^ mice died early during embryonic development due to liver disintegration [5]. Similarly, *Adar2* ^−/−^ mice have been found to be prone to seizures and to die young, and Higuchi et al. have rescued the lethality by mutating the Q/R sites of *GluR-B* mRNA, which were totally edited in normal mice [6]. In addition to the ADAR family, the apolipoprotein B protein family also harbors editing activities, including apolipoprotein B-editing catalytic subunit 1-4 (APOBEC1-4) [7]. The ApoB proteins are primarily responsible for modifying cytosine to uracil (C to U) on the RNA level [8]. Unlike the *Ad*ar1^−/−^ mice, *Apobec-1^−/−^* mice are healthy and viable [9].

It is important to note that RNA editing occurs within a dsRNA structure because ADAR always acts on double-stranded RNAs [10], and the edited inosine in RNA would be read as guanosine (G) by the translation and splicing machineries. Athanasiadis et al. have discovered more than 14,500 editing sites in over 100,000 human mRNAs via computational analysis based on the cluster and double-stranded characteristics of editing sites [11]. Millions of editing sites have been identified due to high-throughput sequencing technology improvement [12]–[14]. However, the data achieved from high-throughput sequencing are heterologous, which means that thousands of false positive sites are also included. Subsequent bioinformatics analysis is crucial for accurate output, and hence, an excellent algorithm is necessary for RNA editing site prediction.

Here, we developed a novel method to predict RNA editing sites based on a random forest method. We conducted a careful feature selection procedure to select a small subset of all features as the optimal feature set to build the model. We found that by using only a subset of features, we can achieve accurate predictions.

## Materials and Methods

### 2.1 Dataset

The RNA-editing dataset used in this study was obtained from the work of Laurent and his colleagues [15]. Laurent et al. sequenced the RNAs and DNAs from wild-type *Drosophila* and RNAs from an ADAR null mutant that lacks the enzyme to mediate adenosine-to-inosine (A-to-I) RNA editing. The DNA data from wild-type *Drosophila* and RNA data from the ADAR null mutant removed the effects of DNA polymorphisms and other non-ADAR artifacts on the RNA-editing sites discovered from RNA data on wild-type *Drosophila*. The sequencing data can be found in the NCBI database (http://www.ncbi.nlm.nih.gov/sra?term=SRP028559).

The training dataset was retrieved from the training samples used to build their Random Forest algorithm-based model, RFss1, as well as for training their sparse partial least-squares (SPLS)- and artificial neural network (ANN)-based models. There were 127 RNA-editing (positive) and 127 non-RNA-editing (negative) samples in the training dataset. The testing dataset was their ‘all but training samples’ dataset used to validate the RFss1 model and the SPLS- and ANN-based models (i.e., all the currently validated sites that were not used for training the models). These two datasets are provided in the **Information S1** in our study. The testing dataset contained 533 positive and 90 negative samples.

The 77 features used were obtained from Laurent et al.'s Table S2, which described the variables used in their Random Forest (RF) algorithm. We performed careful feature selection to identify an optimal feature set to build our final model.

### 2.2 Random Forest

In this study, the RF was employed in prediction model construction. The RF method is a commonly-used classification method containing a number of decision trees. A final classification label was determined based on the class with the most votes from all trees. The RF algorithm has been successfully applied in several bioinformatics studies, such as [16]–[19]. For a detailed description of the RF algorithm, please refer to [20]–[21]. The Random Forest classifier in the Weka 3.6.4 [22] software was employed with default parameters to perform the prediction.

### 2.3 Feature selection

Not all 77 features contributed to the final prediction. A smaller but more effective set should be selected from the 77 features to build the final model. The Maximum Relevance Minimum Redundancy (mRMR) algorithm was used in this study. The mRMR criteria can be used to rank the importance of the 77 features. A feature was deemed more important if it had more relevance to the target but less redundancy among the features themselves. After calculation by the mRMR method, the 77 features were ordered from maximum relevance but minimum redundancy to minimum relevance but maximum redundancy. In the ordered list, a feature with a smaller index indicated that it had a better trade-off between the maximum relevance and the minimum redundancy and was therefore more important. For mRMR method details, please see [16], [23]–[25].

To determine the optimal feature set, the Incremental Feature Selection (IFS) method [16], [23]–[25] was used. Based on the ranked feature list from the mRMR approach, features in the ranked feature list were added one by one from higher to lower rank. A new feature set was constructed when another feature was added. For each feature set, a predictor was constructed and examined. The optimal feature set was obtained when the corresponding predictor yielded the best performance.

The reasons we used the Maximum Relevance Minimum Redundancy (mRMR) and the Incremental Feature Selection (IFS) method are as follows: “mRMR+IFS” allows us to select a compact set of features at very low time cost because the order in which to add features to final feature set in the IFS is determined by the mRMR, which requires low CPU time. However, in the method of backward feature selection and forward feature selection, the order in which to add features is determined by an extended computation to seek the best one, and therefore the time cost increases exponentially. Ref [23] clearly presents comparisons of “mRMR+IFS” with other methods (such as backward feature selection and forward feature selection) on different data sets and notes that “mRMR+IFS” could achieve a good tradeoff between time expense and classification performance [23].

### 2.4 Performance measurements

In this study, we used the 10-fold cross validation method to assess the performance of the prediction model. The complete training dataset was randomly split into 10 equal parts, with each part being used in turn as test data and the remaining 9 parts as training data. This process was iterated 10 times.

The following formulas were used in this study: 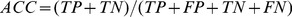
(1)

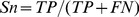
(2)

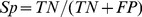
(3)

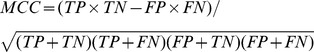
(4)in which TP, TN, FP, and FN denote the number of true positives, true negatives, false positives, and false negatives, respectively. *Sn* and *Sp* represent the sensitivity and specificity, respectively. *MCC* (Matthews correlation coefficient) was used throughout this study as the main evaluator.

## Results

### 3.1 The mRMR and IFS results

**Figure 1:**
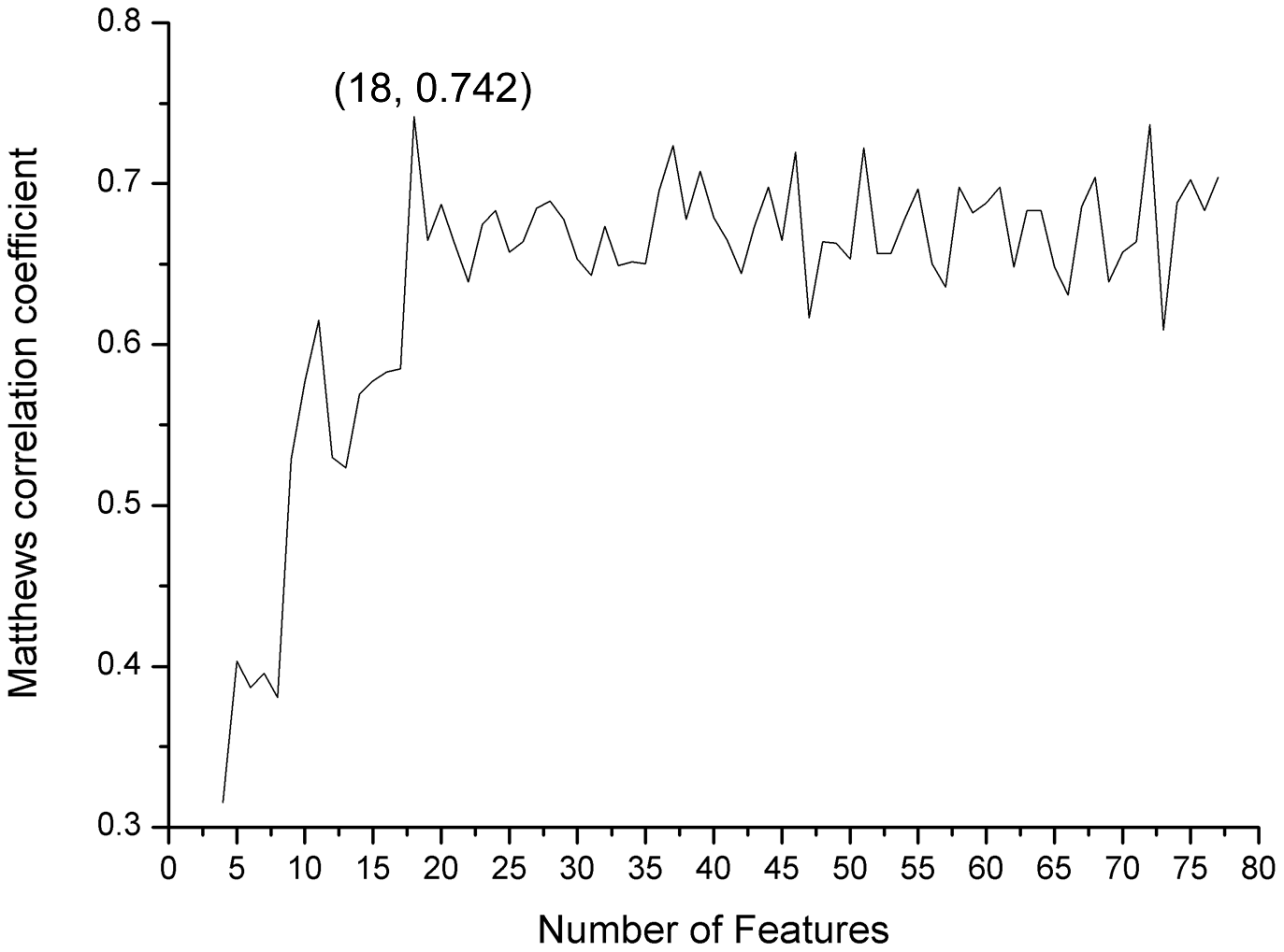
The IFS curves in the training dataset. The plot shows the *MCC* values of the predictors constructed using different numbers of top features selected from the corresponding mRMR table during the IFS process. When the first 18 features were selected, the *MCC* reached its maximum value of 0.7415.

In the training dataset, from the corresponding mRMR table, the ranked 77 features were added one by one to generate 77 different feature sets. Accordingly, we constructed 77 individual predictors. The classification performances are found in **Information S2**. We plotted the predictor *MCC* values in **Fig. 1**. As shown in **Fig. 1**, we achieved the best performance when the top 18 features were selected (with the highest *MCC* 0.741). These 18 features were considered to compose the optimal feature set and were used to construct the final prediction model. The corresponding *Sn*, *Sp*, and *Acc* values for the final model training process are listed in **Table 1**. The accuracy reached a value of 0.866.

**Table 1:** The prediction performance of the final model using 18 features, by 10-fold cross validation.

Dataset	Features	*SN*	*SP*	*ACC*	*MCC*
Training	18	0.945	0.787	0.866	0.742
Testing	18	0.897	0.756	0.876	0.576

### 3.2 The testing results

The final model was tested on our independent testing dataset, and the results are shown in **Table 1**. The accuracy of our model achieved a value of 0.876, which was higher than the accuracy of Laurent's work, 0.87 [15]. We only used 18 features, a subset of the full dataset (77 features). This result suggests that by using only a small feature set, we can achieve accurate prediction.

### 3.3 The optimal feature set

The final model suggested that 18 features were important for the prediction, which are listed in **Table 2**. These 18 features could play significant roles in A-to-I RNA editing. Further analysis of these features in **Table 2** will be given in the following section.

**Table 2:** The 18 optimal features selected in this study and their descriptions.

Rank	Name	Description
1	wt_treads	The total number of reads from the ‘total’ alignments (sense+antisense) (containing G, A, T, C and gaps) in wild-type RNA
2	wt_3AGagap	The ratio of the number of gaps in ‘3AG’ antisense alignments to the total number of reads in ‘3AG’ antisense alignments (containing G, A, T, C and gaps) in wild-type RNA
3	ad_naC	The ratio of the number of C reads in ‘normal’ antisense alignments to the total number of reads in ‘normal’ antisense alignments (containing G, A, T, C and gaps) in ADAR- RNA
4	G3let_tot_rat	The number of G reads from ‘3AG’ and ‘3TC’ alignments (both sense and antisense) divided by the number of G reads from the ‘total’ (sense+antisense) alignments in wild-type RNA
5	wt_3AGsT	The ratio of the number of T reads in ‘3AG’ sense alignments to the total number of reads in ‘3AG’ sense alignments (containing G, A, T, C and gaps) in wild-type RNA
6	wt_3TCsT	The ratio of the number of T reads in ‘3TC’ sense alignments to the total number of reads in ‘3TC’ sense alignments (containing G, A, T, C and gaps) in wild-type RNA
7	ad_3AGsT	The ratio of the number of T reads in ‘3AG’ sense alignments to the total number of reads in ‘3AG’ sense alignments (containing G, A, T, C and gaps) in ADAR- RNA
8	ad_nsT	The ratio of the number of T reads in ‘normal’ sense alignments to the total number of reads in ‘normal’ sense alignments (containing G, A, T, C and gaps) in ADAR- RNA
9	A3let_nG_rat	The number of A reads from ‘3AG’ and ‘3TC’ alignments divided by the number of G reads from normal alignments in wild-type RNA
10	wtnsas_ratG	The ratio wt_nsG/(wt_naG+0.001))
11	ad_3TCsgap	The ratio of the number of gaps in ‘3TC’ sense alignments to the total number of reads in ‘3TC’ sense alignments (containing G, A, T, C and gaps) in ADAR- RNA
12	repeat	1 if the site falls within a region designated as a repeat, 0 if it does not
13	wt_3TCaT	The ratio of the number of T reads in ‘3TC’ antisense alignments to the total number of reads in ‘3TC’ antisense alignments (containing G, A, T, C and gaps) in wild-type RNA
14	wt2adG	The ratio of the number of G reads in the ‘total’ (sense+antisense) alignments in wild-type RNA to the number of G reads in the ‘total’ (sense+antisense) alignments in ADAR = RNA
15	ad_naG	The ratio of the number of G reads in ‘normal’ antisense alignments to the total number of reads in ‘normal’ antisense alignments (containing G, A, T, C and gaps) in ADAR- RNA
16	wt3tA	The ratio of (wt_3AGsA+wt_3TCsA+ wt_3AGaA+wt_3TCaA)/(the number of A reads from the ‘total’ (sense+antisense) alignments) in wild-type RNA
17	wt_3TCsgap	The ratio of the number of gaps in ‘3TC’ sense alignments to the total number of reads in ‘3TC’ sense alignments (containing G, A, T, C and gaps) in wild-type RNA
18	wt_t_AGRL	The average length of G reads for the ‘total’ (sense+antisense) alignments in wild-type RNA

## Discussion

Although RNA editing is reported to be a ubiquitous biological event, and our understanding of such events has increased, our understanding of the mechanism of RNA editing is still far from complete. In our study, we used several features to predict RNA editing sites. These features are applied to two types of RNAs: from wild-type *Drosophila* and from ADAR knockout (ADAR-) *Drosophila*.

Although dozens of features were tested, 18 relatively important features are analyzed below. The first feature is the total number of reads from total alignments in the wild-type RNA (wt_treads). As we know, the RNA editing site annotations are more accurate at deeper alignment depths [26]. The wt_treads is taken as our primary feature to guarantee good data quality. Another group of features used in our algorithm is the gaps-related features, which consist of the ratio of the numbers of gaps in ‘3AG’ antisense alignments (wt_3AGagap), the numbers of gaps in ‘3TC’ sense alignments (wt_3TCsgap), and the numbers of gaps in ‘3TC’ sense alignments in ADAR-RNA (ad_3TCsgap). In addition to substitution, the insertion/deletion of nucleotides such as uracil also plays an important role in RNA editing, especially in the kinetoplastid mitochondria [27]. Nucleotide insertion or deletion could result in gene-encoded reading frame shifts and the creation of complete reading frames in mRNAs that are crucial in gene expression [28]–[29]. In addition, small gaps adjacent to adenosines could also influence the catalytic activities of the ADAR enzyme [30]. Because the main form of canonical editing is the substitution of A with I, where inosine (I) is read as guanosine (G), the characters of G reads are the ideal features for identifying A to I editing sites. In our work, 6 G reads-related characters are used for computation. They are the ratio of G reads in ‘3AG’ and ‘3TC’ alignments compared to the total G reads (G3let_tot_rat), the ratio of A reads in ‘3AG’ and ‘3TC’ alignments compared to the total G reads (A3let_nG_rat), the ratio of G reads in sense alignments compared to the G reads in antisense alignments (wtnsas_ratG), the ratio of G reads in WT RNA compared to the G reads in ADAR- RNA (wt2adG), the ratio of G reads in the antisense alignments compared to the total reads in ADAR- RNA (ad_naG), and the average length of G reads for the total alignment (wt_t_AGRL). As the A to G substitution on the sense strand and the T to C substitution on antisense strand could contribute to the editing sites in our algorithm, the ratio of G reads in ‘3AG’ and ‘3TC’ alignments could identify potential editing sites. Alternatively, a knockout of ADAR in *Drosophila* results in a deficiency of A to I substitution; hence, the G reads produced by substitution in ADAR-RNA represent the background noise compared with the G reads in WT RNA. The length of G reads for total alignments is also included in our algorithm. In the alignment procedures, the mapping read length is critical for alignment accuracy and precision [31]. Similar to the G reads, the ratio of other nucleotides is also meaningful in our computation, such as the ratio of C reads in antisense alignments compared to the total reads in antisense alignments in ADAR- RNA (ad_naC), the ratio of T reads in the ‘3AG’ sense alignment compared to the total reads in the ‘3AG’ sense alignment (wt_3AGsT), the ratio of T reads in the ‘3TC’ sense alignment compared to the total reads in the ‘3TC’ sense alignment (wt_3TCsT), the ratio of T reads in the ‘3AG’ sense alignment compared to the total reads in the ‘3AG’ sense alignment in ADAR- RNA (ad_3AGsT), the ratio of T reads in the sense alignments compared to the total reads in the antisense alignments in ADAR- RNA (ad_nsT), the ratio of T reads in the ‘3TC’ antisense alignments compared to the total reads in the ‘3TC’ antisense alignments (wt_3TCaT), and the ratio of A reads in the ‘3AG’ and ‘3TC’ alignments compared with the total A reads (wt3tA). The above features might influence the mapping of the corresponding reads and the binding of ADAR enzymes to different degrees. Katrina et al. have reported that the editing sites exhibited a 5′ neighbor preference (A≈U>C = G), and ADAR2 exhibited a 3′ neighbor preference (U≈G>C = A) [32]. Based on these findings, we choose the above optimized characters for our training analysis. Another critical feature applied in our algorithm is contig repeats. Previous studies have reported that editing sites were preferentially clustered in the Alu repeats [11], [33]. The canonical editing events of A to I substitutions are dependent on the ADAR enzyme, and the double-stranded structure of the targeted sequence is necessary for this biological process [34]. Including the repeats as a feature in our computation process increased the number of editing sites found.

## Conclusions

As an important post-transcriptional RNA regulation mechanism, RNA editing increases the diversity of gene expression. Understanding the features that affect RNA editing is challenging but fascinating for both experimental biologists and computational biologists because it is essential for accurate genetic manipulation. Based on publicly available datasets, we developed a random forest based method to predict RNA editing sites. Using sophisticated feature selection methods, 18 optimal features were selected. Their prediction *accuracy* and *MCC*, respectively, were 0.866 and 0.741 on the training dataset and 0.876 and 0.576 on the testing dataset. Furthermore, the 18 features provided clues about the mechanism and dominant factors in RNA editing.

## Supporting Information

Information S1 **The complete dataset used in this study, consisting of the training and testing datasets.** There were 127 positive and 127 negative samples in the training dataset and 533 positive and 90 negative samples in the testing dataset.
(ZIP)

Information S2 **The performances of the different predictors constructed using different numbers of top features selected from the corresponding mRMR table during the IFS process.** *Sn*, *Sp*, *Acc*, and *MCC* are given. *MCC* was used as the main evaluator. Upon selection of the first 18 features, *MCC* reached the maximum value of 0.742.
(XLS)
